# Supplemental Review

**Published:** 1821-09-01

**Authors:** 


### Supplemental Review

AND

### QUARTERLY PERISCOPE

OF

### PRACTICAL MEDICINE, SURGERY, &c. &c.

*WITH COMMENTARIES.*

*Paucis libris immorari et innutriri oportet, si velis aliquid trahere, quod in animo fideliter hæreat.* SENECA.

Duo vitia vitanda sunt in cognitionis et scientiæ studio. ***** Alterum est vitium, quod quidam nimis magnam operam conferunt in res obscuras atque difficiles, easdemque non necessarias. CICERO.

1. *Cardiac Diseases.** It is the object of M. Fodéré, in the present paper, to state some facts, and make some observations on diseases of the heart, that may assist the young or inexperienced practitioner in discriminating these dangerous complaints, especially as they take place in *young persons,* whom M. Fodéré's situation of physician to the Royal College of Strasburg, has afforded him opportunities of observing.

*General Observations.* Our author seems to doubt whether the violent political agitations of the last thirty years have done more than increase the *functional* affections of the circulating organ; but, as he has himself shewn that the moral emotions of our nature are continually operating on an organ endowed with the highest degree of animal excitability, there can be no doubt that the structure becomes affected by a long continuance or great degree of these. Such effects of the passions are greatly heightened by intemperance, especially in spirituous liquors, which not only derange the balance of the circulation at the time, but by deteriorating other organs, as the stomach, liver, &c. indirectly and ultimately affect the heart. M. Fodéré hazards a conjecture that climate and locality may have a tendency

* Memoire sur les palpitations et sur l'anevrysme du cœur. Par M. Fodéré. *Journal Compl. No.* 32—34.

to induce diseases of the heart, as we see is the case in respect to certain other organs of the body, as for instance the lungs, thyroid gland, &c. He believes that this class of complaints is peculiarly prevalent in and about Strasburgh. He very properly directs the attention of the reader to the intimate physiological connexion of the heart and lungs. One of the most ingenious anatomists and physiologists of our own country (Mr. Charles Bell) is accustomed to describe these two organs as only two parts of the same apparatus, so mutually dependant are they on one another, and so essentially necessary is each to the grand function of the circulation. If so blended in function, we need not wonder at their participation in disease; and daily observation shews that pulmonic obstruction, and other affections of the respiratory apparatus operate decisively on the functions and even structure of the heart. Dr. Fodéré therefore is not surprized that the question should be agitated whether asthma causes disease of the heart, or diseases of the heart asthma. Our author has frequently observed delirium announce the fatal termination in acute peripneumony; the face being bloated, the cheeks of a violet colour, and the jugular veins pulsating. Dissection, in these cases, shewed the right cavities of the heart dilated and gorged with black blood from the difficulty of forcing this fluid through the lungs. He has always observed the same phenomena at the close of chronic peripneumony and hepatisation of the lungs. In short, in all pulmonic affections we have to dread derangement in the organ of the circulation. Moreover, our author remarks, that during the great period of growth, that is, from ten to eighteen years of age, the blood appears to be carried in the greatest abundance to all the organs, but particularly to the lungs and head. Now if this fluid experiences an obstruction to its free movements in these organs, it is reasonable to expect that the moving power (the heart) must suffer, unless local plethora be relieved by spontaneous hæmorrhage, examples of which will be adduced in the sequel. M. Fodéré has also seen many instances where dyspnœa and symptoms of cardiac disorder have succeeded amputations of the lower extremities, and which he was unable to relieve by any other means than frequent bleeding, and low living.

But besides the various occasional causes of disorders of the heart, there is too much reason to believe that a native or hereditary disposition or peculiarity of construction is often called into action by causes which, in other people, would produce no such effect.

When sympathetic or functional disorders of the heart have their causes at a distance from the central organ, as in the stomach, liver, or bowels, from indigestion, worms, biliary derangements, &c. they generally disappear quickly on removing the causes, unless time has rendered habitual the functional disorder, or begun to alter the texture of the heart. We should be careful, therefore, how we assure our patients that palpitations and other phenomena of morbid action in the circulating apparatus, are purely nervous or sympathetic, however certain we may be that they were so originally; since, as we have just said, time and habit do much mischief. Functional disorders of the heart are more prevalent, in proportion to organic, among the class of literary, sedentary, delicate, and convalescent people, and our prognosis should be modified accordingly. In such classes, a trifling agitation of mind or exertion of body will cause the heart to palpitate, and the arteries to beat as violently as if the patient laboured under inflammatory fever—and we have seen medical men thrown greatly off their guard upon such occasions, having recourse to blood-letting and other antiphlogistic measures, when a little repose only was necessary. In like manner the translation of rheumatism from a joint, or the repulsion of an eruption, will be suddenly followed by violent palpitation of the heart, which as suddenly subsides when the rheumatic inflammation or cutaneous affection is reproduced in its proper place.

The prognosis in diseases of the heart is almost always unfavourable. Palpitation itself, however, especially when the breathing is not much affected, may continue for years, or even for a long life, without much inconvenience. Nevertheless, in general, it is to be viewed with a suspicious eye, for those who have been affected with it from youth upwards, very commonly die before the usual term of human existence. Diseases, whether of function or structure of the heart, are so varied in their symptoms, anomalous in their characters, and irregular in their progress, that nothing but the study of numerous individual cases can fortify us against the exigencies of practice. We therefore intreat our readers' patient attention to the following cases, the histories of which we shall abridge as much as possible, or consistent with perspicuity.

*Case* 1. On the 17th November, 1817, a young eleve, sixteen years of age, was brought to the infirmary of the Royal College of Strasburgh, with the following symptoms:—the face flushed—the eyes sparkling—skin hot and dry—the chest stuffed—pains in the head. The pulsation of the temporal, carotid, and subclavian arteries was visible—and the action of the heart was so violent that it was audible, and raised the clothes at each stroke. The pulse was full and hard—the appetite keen. The youth was of a full habit; and had been subject to nasal hæmorrhages. Viewing the cardiac affection as depending on growth, and on a want of harmonious balance between the vascular, respiratory, and nervous systems, M. Fodéré prescribed a copious general bleeding, warm pediluvia, absolute rest, aqueous drink, and diet almost purely vegetable. These means being insufficient, and the motions of the heart and arteries continuing too violent, a grain of digitalis was ordered four times a day. This medicine soon reduced the vascular action; but a train of nervous symptoms succeeded, which obliged him to intermit the fox glove, in substitution of which nitre and camphor were given. From this time the patient grew daily better, and by Christmas he was able to leave the infirmary. He had several relapses similar to the above, at various intervals. They were all relieved by the same means; and when M. Fodéré saw the patient last, on the 18th September, 1820, nothing remained but a slight inordinate action of the heart, of which the youth himself was unconscious.

*Case* 2. On the 12th March, 1818, Master H———, aged 17 years, presented himself at the Infirmary, with his neck swelled, and complaining of pain in his ears, which were very red. He had no palpitations. These symptoms were removed by low diet, diluents, and pediluvia. On the 26th May a relapse; but now there was violent pain in the occiput, considerable hæmorrhage from the ears, enormous palpitation of the heart, hard, full pulse. Bled largely from the arm, which was repeated next day, rigorous abstinence from food, lemonade for drink, pediluvia daily, digitalis four times a day. On the 11th June he was able to leave the hospital, but still there was some palpitation of the heart. On the 7th August, of the same year, another relapse, requiring a repetition of the above measures. After this he was put upon a most rigorous regimen, abstinence from animal food, wine, beer, liqueurs, coffee, or any exertion of body or mind that might accelerate the action of the heart. He gradually, though slowly, recovered."

We shall pass over several cases of palpitation which were symptomatic of rheumatism, scorbutus, chorea, and nervous mobility, all of which were easily cured by the appropriate remedies for such disorders.

*Case* 3. The *eighth* case was that of a youth, 14 years of age, who had been treated by an eminent physician, for more than twelve months, for organic disease of the heart. M. Fodéré, on placing his hand on the region of the heart, perceived there the greatest disorder of function. The face, excepting the centre of the cheeks, was pallid, the strength gone, the eyes dull, and he complained of anomalous pains in various parts of the body. After a minute investigation, M. Fodéré suspected onanism, and the youth, on being closely pressed, confessed it. It was now decided that the palpitations of the heart were nervous, and owing to debility. The pernicious custom was guarded against—the bowels were cleared with calomel—digitalis and antispasmodics were combined, and the cure was completed by decoction of bark.

We shall omit the ninth case, which is of minor importance, and proceed to a rather minute account of the tenth; because we have unfortunately seen several of a similar description, and in which our hopes had been buoyed up too much by the fallacious appearances which cardiac diseases so frequently assume.

*Case* 4. M. Fodéré was consulted, about two years ago, respecting a young lady, 18 years of age, whose father was gouty, and whose sister died of tubercular consumption. This young lady was tall and large of her age, with considerable muscular force. Her pulse was slow, and indicative of languor in the vital organs—the sexual and other functions, however, were healthy. She complained to our author, as a thing of very trifling importance, in her estimation, that, for two years previously, she felt, occasionally, a pain in the left side of the chest, and stretching to the spine, which was sometimes so acute as to oblige her to remain motionless, for a short time while it lasted. On requesting to examine the thorax, M. Fodéré found the young lady bound up with stays drawn so tight that he could not introduce his fingers between them and the chest. When unlaced, our author perceived, with horror, the heart beating, with an undulating stroke, from the clavicle to the false ribs of the left side. He pronounced it, at least, in his own mind, a passive aneurism of long standing, the rupture of which he could not calculate the epoch of. He wished to avoid having any thing to do with the case, but was pressed by the family to undertake its management. He knew not well what to do; but viewing the languor of the circulation, and finding that the young lady had some indication of scurvy, he prescribed vegetable-antiscorbutic drinks, which soon had the effect of quickening the pulse, and lessening the palpitations. Encouraged by this, our author combined the syrup of cinchona with the above, and in a little time he was surprized to find the symptoms of cardiac disease disappear, and the patient get apparently well. The young lady was subsequently seized with a biliogastric fever, during the continuance of which, the heart did not manifest any unusual derangement of function, a circumstance that confirmed M. Fodéré in the hopes of a perfect cure of that disorder. The patient recovered from the fever, and our author discontinued his visits. In a few days, however, a messenger came running to M. Fodéré to say the young lady was expiring. He ran to her, but she was dead! She had been coming down stairs to dinner, apparently in perfect health, when the thread of life was thus suddenly snapped. Dissection shewed the right ventricle passively dilated, thin, and torn, the pericardium being filled with black blood. The pulmonary artery was greatly dilated, and the lungs partially diseased in structure.

Such is the treacherous nature of diseases of the heart. The most terrible *organic* changes will frequently appear to be quite cured; while the practitioner feels mortified at the unnecessary alarms into which he had thrown himself and the friends of the patient. On the other hand, nervous *functional* disorder will mask itself in the most threatening forms, and harrass physician and patient, for months or even years, with the fears of momentary dissolution, and, after all, vanish unexpectedly, and the patient regain perfect health. A knowledge of these things will render a physician cautious in his prognosis—the more experienced he becomes indeed, the more averse will he be to decide positively on the fate of his patient. He will, however, become more judicious in his treatment, and less liable to do mischief by pursuing unnecessary or improper measures.

Lancisi, de Haen, Frank, and many able physicians, have entertained an opinion, that cardiac diseases were occasionally hereditary. There can be no doubt, indeed, that many people are born with that structure of the heart and vascular system which, under the application of very feeble exciting causes, (or even without these,) is very apt to fall into disorder. Why should not this be the case with the heart, as well as with the lungs? Frank observes that it is not unusual to find aneurism of the heart in the fœtus, an example of which is seen under the word "*vie du fœtus*," in the Dict. des Sciences Medicales.

When M. Fodéré had charge of an hospital on the borders of the Mediterranean, during the late war, he met with many cases of disease of the heart among the peasantry, who are exposed to great and sudden atmospherical transitions, which derange the functions of the skin, and produce violent oscillations in the tide of the circulation, giving origin, he rationally supposes, to functional and organic disorders of the heart and great vessels.

*Case* 5. In the spring of 1806, M. Fodéré was called to the assistance of a young female peasant in the village of Gignac, whom he found emaciated, and complaining of constant uneasiness, pricking sensation in the skin, want of sleep, head-ache, and a rushing noise all over the body. She had menstruated but once, and that twelve months previously; since which, she had been subject to nasal hæmorrhages, and sometimes spitting of blood. The pulse was frequent and irregular. The same of the heart, but with violent palpitation both in the chest and abdomen—it was these pulsations that caused so much inquietude. Our author was much embarrassed as to the mode of treatment which he ought to pursue; but thinking that the suppressed menstruation might make part of the cause of these strange phenomena, he ordered leeches to the sexual organs, semicupia, diluent drinks, and digitalis. Being ordered to another part of the country, M. Fodéré did not see the patient for twelve months; but was then agreeably surprized to find his patient in good health, having persevered a considerable time in the measures abovementioned.

Another case, somewhat similar, is related by our author, and then he concludes with the detail of a melancholy case, of which we shall present the prominent features.

*Case* 6. In the beginning of 1810, M. Fodeéré was consulted by a merchant, Mr. S——, of Marseilles, aged 45 years, who appeared greatly out of breath in walking up a street to M. Fodéré's house. On attentive examination, our author concluded that there was an aneurism of the heart, or of the arch of the aorta. There was enormous palpitations through the whole chest, extending to the left arm, the muscular power of which was much affected. The two first ribs, as well as the clavicle of that side, were bulged out, and there was an evident engorgement of the liver, the cæliac artery pulsating nearly as much as the aorta in the chest. The face was flushed and spotted—the urine scanty and lateritious—the pulse full, frequent, and intermittent. The appetite and generative functions were unimpaired. Mr. S. coolly stated to our author that his father died by the bursting of an aneurism at the age of 48, and that he knew he had the same disease. All he wished, or at least expected, was to keep the malady at bay till he should arrive at the age of 50 years. Hitherto the patient had deprived himself of no pleasure—not even that of hunting; but our author persuaded him to adopt the rigid regimen and quietude of body so necessary in organic diseases of the heart. He was also bled from the arm, had leeches applied to the anus, and he had proper medicines for reducing the abdominal congestion. By adoption of these measures, he was astonishingly improved. The abdomen became flaccid, the urine more abundant, the palpitations less severe, even the bulging out of the ribs and clavicle subsided to their natural level. M. Fodéré now quitted Provence, but happened to pass through it again four years afterwards, and called on his patient. But, alas! all the bad symptoms had returned, and the following year, Mr. S. died suddenly, as his father had done before him, and in the 50th year of his age, the goal at which he seemed to aspire!

*Reflections and Conclusions.* 1. M. Fodéré asks whether we can hope to cure aneurism, even when taken in the earliest stage of its progress? He seems disposed to doubt our power, in this respect, because few, if any, can keep a perpetual curb on their appetites and passions, without which a cure cannot be expected. Every one knows that to check a disease of this nature, we must reduce the quantity of circulating fluids, and diminish their stimulating properties. All temporary increase of the motion of the fluids must also be guarded against by avoiding all exertions of body, and perturbations of mind. M. Fodéré justly observes that we should be anxious to remove palpitations of the heart in all cases, because a continuance of this inordinate action very frequently leads to organic disease. We should therefore carefully investigate the cause of this phenomenon, and endeavour to ascertain whether it depends on want of freedom in the respiratory function, on worms, gastric derangement, affections of the kidnies or bladder, spasmodic tendency, debility, exhaustion, or mere nervous irritability. Much of our success in the treatment will necessarily depend on our accuracy in ascertaining the occasional cause.

2. Can we hope, says M. Fodéré, to procrastinate long the fate of actual and confirmed aneurism of the heart or aorta, allowing that the patient adopts our precepts to the full? Our author thinks, and in this supposition he is supported by De Haen, that a patient, after aneurism of the heart is fairly formed, cannot survive more than five years, however guarded he may be in regimen, and however well he may be attended medically. Two or three instances have come within our own observation, where aneurismal enlargements of the heart, both active and passive, have continued longer than the aforesaid period, before they terminated fatally. At the same time, we believe that, in a majority of cases, death will take place before the expiration of five years from the commencement of actual enlargement of the organ of the circulation.

3. Dr. Fodéré considers, from attentive observation of facts, that of all kinds of drink for those afflicted with this dreadful disease, as well as all chronic inflammations, *whey* is the best. Of aliment, he thinks *milk* is least stimulant, and yet sufficiently nutritive. Of the powers of digitalis, in lowering the tone of the circulation, and relieving the heart, our author entertains a very favourable opinion, not from using it, in routine, but from carefully watching its effects.

Finally, M. Fodéré properly remarks that it is of great consequence to distinguish active from passive aneurism of the heart, inasmuch as the *former* requires a *decided* antiphlogistic treatment, which, in the same degree, would be injurious in the latter form of the disease. The pulse will, in general, lead to the diagnosis; but the whole phenomena of the complaint should be accurately investigated, in order to come to a just decision on the diagnosis.

2. *Ulceration of the Cartilages.** This rare disease begins with rheumatism, and like it also, attacks several joints in succession. No treatment seems to stop its progress, and at length it ends in complete anchylosis. The time, which this process took up in Mr. Mayo's cases, was about two months.

We recollect two sisters, who were afflicted with this disease. In one of them all the joints were anchylosed, excepting the shoulders, before we saw her; even the spine had not escaped. She lay in bed in nearly the same position, in consequence of the complaint, from fifteen to twenty years, at the end of which time she died. We saw the other soon after the disease commenced. It began in this instance in the phalanges of the fingers, most of which were either immoveable, or in a state approaching to anchylosis. The appearance of the joints during the progress of the disease resembled gout, excepting that there was less external vascularity. Leeches, blisters, mercurials, sea-bathing, soda, potass, &c. were tried in vain. The result of the latter case we do not know, but we have reason to think that the disease did not extend farther than the extremities. Both of these ladies were married and had the scrophulous diathesis, and one of them had a family of children.

3. *Extra Uterine Conception.*† Among the aberrations of Nature (which fortunately are rare in the generative process) none are so truly deplorable as extra-uterine conception. The following case presents many points of interest to the pathologist and physiologist, which we deem it proper to record.

" Dominica, Mirone, 23 years of age at the epoch of her marriage, was, before that period, subject to vomitings, fluor albus, and hysterical affections. Seven years after marriage she became a mother, at the regular time. She had afterwards a seven month child. In December 1817, she became pregnant for the third time, and this pregnancy lasted sixteen months. In the second month she had some shew of the menses—in the third month she experienced pains in the lower belly, and a considerable serous discharge from the uterus. From the fourth to the thirteenth month she menstruated irregularly. In the fifth month she felt the fœtus; in the seventh very distinctly. These movements continued till the thirteenth

* Mr. H. Mayo, *Med. Chir. Trans.*

† Memoria sopra una gravidanza estra-uterina. Par Laurent Rizzo.

month, principally in the left side. The breasts became enlarged in the sixth month, and furnished some milk in the seventh. In the twelfth month the breasts subsided to their original dimensions. About this period the woman felt what she conceived to be labour pains. In the thirteenth month a uterine hæmorrhage took place, and lasted some days. At different periods of the extra-uterine pregnancy she suffered dreadful colicky pains—frequently she had fever, and bilious vomitings. In the sixteenth month she was seized with acute fever, and died with symptoms of peritonitis."

*Dissection.* On opening the abdomen, Dr. Rizzo found the peritoneum inflamed, and some gangrenous spots on it. A large and oval body was discovered in the abdomen, containing a fœtus enveloped in its membranes which were gangrened and burst. Two hydatids were attached to the chorion, containing a yellowish fluid. The chorion had contracted adhesions with all the neighbouring parts, especially the epiploon, colon, posterior face of the uterus, &c. The placenta was attached to the peritoneum covering the vertebral column. The situation of the fœtus was that of a natural pregnancy. The uterus was perfectly sound and natural. The left ovary presented nothing remarkable—the right one was obliterated.

4. *Neuralgia.** This distressing disease becomes more common every day. It would seem as if the progress of civilization and refinement has a more than proportional effect on the *nervous system* of the human constitution, rendering it more irritable and morbidly sensible to the various agents around us, and consequently engendering a host of diseases to which our forefathers were but little subject, comparatively speaking. The following case is interesting and instructive.

A lady, 57 years of age, of strong constitution and good health, was bitten by a little girl who was delirious in fever. The wound was on the back of the second phalanx of the little finger of the left hand. No attention was paid to the circumstance; but, in a few days, pain commenced in the finger, spreading successively to the hand, forearm, and elbow, in the line of the cubital nerve. The wound was cauterized at a white heat, so as to destroy the nervous filament, but this measure produced no benefit, and the

* Observation d'une Névralgie Anomale. Par le Docteur Feron. *Journ. Comp. May* 1820.

neuralgia ascended to the armpit, with increasing violence. Fomentations, fumigations, and large blisters were ineffectual, as were also embrocations, narcotics, the warm bath, &c. It was curious that each time she went into the bath she experienced a sensation of excessive *coldness* throughout that half of the body corresponding with the diseased upper extremity, and an intense *heat* in the opposite side. The equilibrium became restored as soon as she left the bath. Two applications, of fifty leeches each, to the arm, produced a temporary remission of the pain. The patient now experienced a sensation of tightness and stuffing in the chest, to which succeeded a violent cardialgia with vomiting of every thing taken into the stomach. These symptoms lasted six months.

It is to be remarked that the paroxysms of pain always commenced in the wounded finger, which induced the patient to request amputation of the joint. About this time there occurred, for a few days, a remission of the symptoms, after which, a new train ensued. The vomitings and cardialgia ceased entirely, (the neuralgia of the arm remaining as before,) but a copious diarrhœa took their place, and continued for two months. The neuralgia of the digestive organs now shifted to the uterine system, producing violent pain in the hypogastric region, and suppression of the menses.

M. Feron now applied the moxa near the elbow for fifteen minutes, which had an astonishing effect. The pain in the arm ceased—three days afterwards the chest became free —the diarrhœa disappeared—the appetite and embonpoint finally returned. This was in May; but, unfortunately, in October following, the neuralgic affection of the arm re-appeared, together with the other constitutional symptoms before enumerated, and with the addition of an acute pain in the meatus auditorius externus. A new moxa was burnt on the arm, but not with the same success as before. It only caused a considerable mitigation for a time. The complaint is now periodically exasperated on certain days, and has continued altogether for fourteen months. Time or a change of climate is looked to as the principal hope of cure or alleviation.

In the 4th number of the Revue Medicale, M. Bousquet has made some pertinent reflections on this case. He particularly, and with reason, asks why M. Feron did not repeatedly apply the moxa, seeing that it entirely suspended the complaint for some months, in the first instance, and mitigated it in the second? Baron Larrey has had much success with this measure; but he does not content himself with one or two applications of the moxa; he burns it ten, twelve, or more times, and follows the pain wherever it takes up its domicile. In neuralgic affections, M. Delpech destroys the nerve by the actual cautery, and when the eschar separates, he permits not the wound to heal, but keeps up a long-continued suppuration.

5. *Oil of Croton.* A good deal of interest has been lately excited among the profession respecting this *new* and curious substance, a single drop of which produces copious and sometimes violent purging. We have been favoured with a communication from John Flemming, Esq. M. P. pointing out accounts of this medicine published by preceding authors, and also by himself, affording another instance of the adage that there is "nothing new under the sun."

Murray, in the 4th volume of his Apparatus Medicaminum, page 149, gives the following botanical description of the plant from whose seeds the oil is obtained.

"*Croton Tiglium*, foliis ovatis glabris acuminatis, caule arboreo, Linn, *Sp. pl.* p. 1426. Cadel-Avanucu, *Hort. Malab. Tom.* 2, *p.* 61. Rumphius also describes the plant and oil, in his *Herb. Amboin. Tom.* 4, *p.* 98.

We shall quote the following passage from Rumphius:—

"Sed tota stirps, potissimum tamen folia, valde acris, ita ut os, labia, fauces inflammatæ intumescunt, et ardor usque ad anum percipiatur. Radix granis mitior. Olim grana per totam Indiam Orientalem Crebro in usu fuerunt *ad lympham hydropicorum per alvum præprimis eliminandam*, in iis vero, quorum ventriculus debilis, simul emesis subsequuta. Fortioribus bina grana sufficerunt, aliis granum unum cum semisse. Variis aliis in morbis, *in quibus purgantia fortiora opportuna videntur*, ista in India adhibent. *Et hanc quidem acrimoniam oleo ipsi seminis inesse*, tam ex dictis, quam inde apparet, quod olei ex siccis granis expressi *gutta una* cum canariensi vino capta, vulgare apud chirurgos in India degentes purgans constituerit."—*Rumph. Tom.* 4, *p.* 98.

Another writer observes that the oil of croton rubbed about the umbilicus purges in the same manner as when taken internally.

In the 11th volume of the Asiatic Researches, Dr. Fleming has published a valuable paper on the Indian medicinal plants and drugs, in which the croton tiglium is thus noticed:—

"The seeds of this plant were formerly well known in Europe, under the names of *grana tiglia*, and *grana molucca*. They were employed as hydragogue purgatives: but, on account of the violence of their operation, they have been long banished from modern practice. For the same reason they are seldom used by the Hindû practitioners, though not unfrequently taken as purgatives, by the poorer classes of the natives. One seed is sufficient for a dose, being rubbed with a little rice gruel, or taken in a bit of plantain fruit."*

We think the medicine in question is deserving the attention of the faculty at large.

6. *Acute Inflammation of the Medulla Spinalis.*† Affections of the spinal brain have, of late, much occupied the pathological physician, and therefore we deem it a duty to bring every detached fact, bearing on an investigation so recently commenced, before the profession as early as possible.

*Case* 1. Mary Brisset, 27 years of age, of nervous temperament, but hitherto enjoying good health, was accused (wrongfully) of having committed a theft. The menses were flowing at the time, but became suddenly suppressed. She was thrown into a state of the greatest mental agitation and despondency in consequence of the false charges against her moral character. On the third day she was found in a state of complete abolition of the functions of sense and intelligence. Taken to the Hotel Dieu, the stupor disappeared in about six weeks, but fatuity continued; on account of which she was transferred to the Salpetriere, on the 18th August, 1818, presenting the following symptoms:—countenance wild— difficulty of articulating words—answers slow, and confused—debility, but not paralysis of arms and lower extremities—listlessness and indisposition to motion—sometimes paroxysms of anger and impatience—the vital functions perfectly healthy. These symptoms scarcely varied during fifteen months—excepting a great increase of corpulence. On the 15th January, 1820, she was seized with convulsions, followed by foaming at the mouth, turning up of the eye-balls, grinding of the teeth, tetanic closure of the jaws, profound stupor, convulsive motions of the trunk, immobility of the limbs, pulse full, frequent, and irregular, respiration short, embarrassed, and precipitate, involuntary discharge of fæces—clammy, rank perspirations. After three days passed in this state, the patient died.

*Autopsia.* Skull thick, and its vessels injected—superior longi-

* Some cases of tic douloureux have lately been relieved, and even removed, by a drop or two of oil of croton applied on the tongue. The effect on the nerve was almost instantaneous.—*Ed.*

† Pinel, fils.

tudinal sinus gorged with blood—tunica arachnoidea presenting marks of chronic inflammation, viz. thickening of its texture, layers of coagulable lymph, serous effusion, and adhesions. Cerebrum and cerebellum presented nothing remarkable. The spinal canal was opened with great care, and the coverings of the spinal brain appeared sound. These being slit open the whole length of the vertebral canal, a pultaceous disorganization of the medulla itself was easily recognized, beginning at the fourth cervical vertebra, and extending to the first lumbar. Throughout this whole space the medulla was reduced to a kind of bouilli, yellowish, semi-fluid, and inodorous. Towards the lumbar region the medulla resumed its ordinary consistence; and *there* it was bathed with a reddish serosity. The thoracic and abdominal viscera appeared remarkably healthy, excepting that the inner surface of the stomach was somewhat red.

We have not been particularly edified by M. Pinel's remarks on this case. The most interesting pathological fact is, the *physical* effect of *moral* causes. The sudden suppression of the menses, from mental emotion, was soon followed by disorder of other and important parts.

*Case* 2. Felicia Lepoigny, of sanguineous temperament, menstruated at 11, and enjoyed good health till her 15th year. At this time being frightened by the entrance of the Russian soldiers into the village, and some attempts by one of them on herself, she was seized with a paroxysm of epilepsy, which returned, with shortening intervals, until her intellectual faculties gave way, and she was conducted to the Salpetriere in 1816, in a state of complete idiotism, complicated with epilepsy, attacking her every four or five days. In this state she continued, without any material alteration, for four years. In January 1820, a succession of dreadful paroxysms of epilepsy and convulsions terminated her wretched existence.

*Autopsia.* Dura mater strongly adherent to the skull, especially in the right parietal fossa—tunica arachnoidea healthy, but rather injected—substance of the brain unaffected—excepting turgescence of its vessels. The cerebellum was rather soft but sound. The spinal brain being laid open its whole length, presented a great turgescence of its venous system. Towards the dorsal region a disorganization, similar to that recorded in the preceding case, was found in the medullary substance; extending from the plexus of nerves going to the arms, as far as the lumbar region. The thorax and abdomen presented no organic lesion.

As acute inflammation of the spinal marrow is a disease little known, M. Pinel endeavours to ascertain what are the principal symptoms by which it is characterized. These he thinks are 1^mo. annihilation of the nervous functions; 2^ndo. convulsive motions of the trunk; 3^tio. evening accessions of fever. *Complete* abolition of the nervous functions happens but in very few cases. Hæmorrhages in both hemispheres, or general softening of the brain can alone produce such an effect. But as in the brain, hæmorrhages and softenings are usually confined to a hemisphere, so the symptoms attending such states are never so general as where the spinal marrow is affected. The experiments of Cuvier, Le Gallois, and Philip, prove the importance of the vertebral brain, and may give us some idea of the effects which disorder of its structure may be expected to produce on the nervous system.

Our author thinks that convulsive motions of the trunk, and annihilation of voluntary motion, are among the most certain signs of acute phlogosis of the medulla spinalis. He conjectures also, that those paralytic affections of the extremities following apoplexies are owing to some compression or some sympathetic affection of the spinal nerves. The evening accessions of fever prove that this disease, in the instance quoted, was of the acute species—probably epilepsy is the result of the chronic form of the medullary phlogosis. But our author is leading us into the region of hypothesis or conjecture, and we have only to remark, in the language of Sydenham—" nobis vero qui non ultra quam res ipsa loquitur sapere audemus, perinde est, an hæc, an alia aliqua hypothesis phenomena rectius solvat." Cap. iv.

7. *Acupuncture.** Of the remedial measures which have been imported into Europe from the Asiatic and ultra-gangetic nations, shampooing, nose-making, moxa-burning, and acupuncture, are the most distinguished. When we consider the great antiquity of civilization among the Chinese and Hindoos, we cannot but conclude, that observation and experience must there have brought to light many important remedies, of which, however, we are almost entirely ignorant. Mr. Wafer, the surgeon of Dampier, relates that the Darien Indians employ a kind of acupuncture, for the purpose of local blood-letting.

" The patient is taken to a river, and seated upon a stone in the middle of it. A native, dexterous in the use of the bow, now shoots a number of small arrows into various parts of the body. These arrows are prepared purposely for this operation, and are so con-

---

* A Treatise on Acupuncturation; being a Description of a Surgical Operation originally peculiar to the Japanese and Chinese, and by them denominated Zin-King, now introduced into European Practice, with Directions for its Performance, and Cases illustrating its Success. By James Morss Churchill, Member of the Royal College of Surgeons in London. Octavo, pp. 86, and plate of instruments. London, 1821.

structed that they cannot penetrate beyond the skin, the veins of which, opened by the puncturation, furnish numerous streams of blood, which flow down the body of the patient." P. 8.

It is very remarkable that this practice is common also in the Society Islands and at Otaheite, as we have been informed by a gentleman who visited those places with Vancouver, and described to us the process, almost in the words which Mr. Churchill uses. Leaving this curious coincidence between two widely distant and uncivilized tribes to the consideration of the naturalist or historian, we may remark that the process alluded to has no affinity to acupuncture as practised in the East, and now in Europe. Notwithstanding the boasted efficacy of the measure, according to the Japanese and Chinese, it did not attract general attention in Europe. In its favour, however, may be enumerated Ten-Rhyne, Bidloo, Kæmpfer, and Vicq-d'Azyr, but none of these had personal experience of the operation. Of late years several practitioners in France have performed acupuncture, and some favourable reports have been made in the Journals. Mr. Churchill's attention was lately directed to it by his friend Mr. Scott, of Westminster, who, he believes, was the first who performed the operation in England. The success which Mr. Churchill observed in Mr. Scott's practice, led himself to its adoption; and the result, he observes, warrants a recommendation of the measure in the strongest terms.

The method of performing acupuncture is simple and easy to any one possessing common anatomical knowledge. It is only necessary that the operator should avoid the course of large vessels, nervous trunks, and the tendons of muscles—though it is not proved that the *latter* sustain any injury from the puncture of the needle. The operation as practised in Japan, is thus described in a historical work.

" 'The place made choice of for the puncture, is commonly at a middle distance between the navel and the pit of the stomach, but often as much nearer to, or farther from, either as the operator, after a due scrutiny, thinks most proper; and in this, and the judging rightly how deep the needle must be thrust below the skin, so as to reach the seat of the morbific matter, and giving it a proper vent, consists the main skill of the artist, and the success of the operation is said to depend. Each row hath its particular name, which carries with it a kind of direction, with regard to the depth of each puncture, and the distance of the holes from each other, which last, seldom exceeds half an inch in grown persons, in the perpendicular rows, though something more in those which are made across the body, thus,

. . .

. . .

. . .

" 'The needles which perform the operation are made, as was hinted at first, either of the finest gold, or silver, and without the least dross or alloy. They must be exquisitely slender, finely polished, and carry a curious point, and with some degree of hardness, which is given by the maker by tempering, and not by any mixture, in order to facilitate their entrance, and penetrating the skin. But, though the country abounds with expert artists, able to make them in the highest perfection, yet none are allowed, but such as are licensed by the emperor.' " 17.

It would seem, from the imperfect accounts that have reached us, that the Asiatics employ acupuncture chiefly in diseases of the abdominal cavity and viscera, such as colic, tympany, &c. but it is not in those diseases that our author has experience of the operation. The Indians themselves, however, extend the measure to most cases of cephalalgia, comatose affections, ophthalmia, &c. They puncture the chest, back, abdomen, &c. for affections of those parts, and also as a cure for dysentery, dyspepsia, histeria, &c. It is only in "local diseases of the muscular and fibrous structures of the body," that Mr. Churchill has hitherto employed acupuncture, but he means to extend its use when opportunities occur. He considers the operation as inapplicable or injurious in diseases of an inflammatory character—a distinction observed by those who have practised this measure. M. Berlioz, of Paris, appears to have employed this curious means of relief pretty extensively, in local pains of a nervous character, and with much success. Speaking of the diseases to which acupuncture is applicable, M. Berlioz remarks—

" 'Vague and wandering rheumatism sometimes attacks the external muscles subservient to respiration; the patient is obliged to remain motionless; every motion of the trunk compels him to cry out: a deep inspiration is very difficult, and coughing occasions such cruel pains, that expectoration is impossible. Acupuncturation dissipates instantly this state of distress, and renders to the muscles their full liberty of action. In the space of one or two minutes, a patient whose sufferings drew from him tears, exclaims he is quite cured.' " 29.

These observations are corroborated by the experience of Dr. Haime of Tours, who has lately published an interesting memoir on acupuncture, in the 13th volume of the "Journal Universel des Sciences Medicales." We shall quote a case as an example.

" A woman had suffered for several days with wandering rheumatic pains, which continued daily to increase in violence; there were, however, at all times fixed pains in the shoulder and in the right arm, which acquired such a degree of intensity by intervals, that the patient could not refrain from crying out. She was in this state when she came to consult me: finding, however, neither alteration in the pulse, nor increase of heat, nor redness of the skin, nor tension, nor swelling in the part affected, I considered the case to be simple rheumatalgia, and passed the needle in the middle of the arm, between the fibres of the triceps brachialis muscle; the place designated by the patient as the seat of the pain. The pain was driven into the fore arm, and the second puncture caused it to descend into the hand, and a third being made in this part, caused it totally to disappear, and the patient said with delight and astonishment, she was cured; and was so satisfied with this treatment, that she spoke of it to every body." 36.

We shall now proceed to notice the cases which occurred in Mr. Churchill's own practice.

The *first* was a bricklayer, ætat. 30, who came to Mr. C's house supporting himself by a stick in one hand, and resting the other against the wall.

"The body was bent at nearly right angles with the thighs, and his countenance indicated acute suffering. He had been attacked, he said, three days before, with darting excruciating pains in the loins and hips; every motion of the body produced an acute spasmodic pain, resembling en electric shock; and the attempt to raise the body to an upright position was attended by such insupportable agony, as obliged him to continue in this state of flexion rather than encounter it by altering his position. There was no more constitutional disturbance than was to be expected from three days and nights of constant pain; the pulse was a little quickened, and the tongue white, but I attributed this derangement to the irritation set up by the pain and loss of rest. I directed him to place himself across a chair for support during the operation, and I immediately introduced a needle of an inch and a half in length into the lumbar mass on the right side of the spine; in two minutes time I observed that he seemed to rest the weight of his body more on his limbs, and in the next instant, without any enquiry being made, he observed, that he felt his limbs stronger from the "pain having left his hips." He next plainly indicated that the disease was lessened, by raising his body; from which he only desisted, by being desired to remain at rest, through fear of the needle being broken. The instrument having remained in its place about six minutes, the patient declared he felt no pain, and could, if he were permitted, raise himself upright; it was then withdrawn; the man arose, adjusted his dress, expressed his astonishment and delight at the sudden removal of his disease, and having made the most grateful acknowledgments, left the house with a facility as though he had never been afflicted." P. 49.

The second case was a young man employed in a timber merchant's yard, who, whilst in the act of lifting a very heavy piece of mahogany, was seized suddenly with a violent pain in the loins. The weight fell from his hands, and he was incapable of raising himself. He was immediately cupped and blistered on the part; but two days passed without much relief.

"On the third day the opertion of acupuncturation was performed upon the part of the loins pointed out as the seat of the injury, which, as in the former case, dissipated the pains in five or six minutes, and restored the motions of the back. He returned, however, the next day, with the same symptoms as at first, but in a mitigated degree. A needle was now passed to the depth of an inch on each side of the spine, which, as I expected, terminated the disease in a few minutes, and it was with pleasure that I understood the next morning, that the man had gone to his usual employment." 51.

We must pass over the other cases detailed by our author, in order to state some particulars of the effects of acupuncture on Mr. Scott, the gentleman who first introduced the operation into England. Mr. Jukes, of Westminster, received an urgent message from Mr. Scott, to visit him immediately. Mr. J. found him in bed, and with a countenance expressive of much anguish, having suffered, for three days, from severe pain in the loins, which he attributed to a sudden transition from a warm room to a foggy nocturnal atmosphere.

"Within the last 12 hours it had acquired such a degree of violence that even respiration was insupportable, except the body were fixed in such positions as permitted the least possible motion. An attempt to resume the erect posture, produced violent spasmodic action of the muscles of the back, which appeared to be communicated by sympathy to those of the abdomen and chest, impeding respiration with a convulsive effort; nor could any motion of the body be made without producing this distressing effect. Neither fever nor general derangement was present; the secreting organs of the body properly performing their function, proved the external locality of the disease. In this state of things, acupuncturation presented itself to us as likely to afford relief, and it was therefore immediately resorted to.

"'I applied an exhausted cupping glass upon the integuments, opposite to the second lumbar vertebra, and midway between this bone and the edge of the latissimus dorsi muscle, which was the part referred to as the most concentrated spot of the disease. As soon as a needle had penetrated to the depth of an inch, a sensation arose, apparently from the point of the instrument, which the patient described as resembling that which is produced by the passage of the electric aura, when elicited to a metallic point, diffusing itself at first to some distance around the part, and then extending itself up the side to the axilla. This sensation continued to be felt for the space of a minute, when a violent pain struck into the right iliac region, immediately above, and corresponding with the line of, the crista of the ilium. No pain was now felt in the back, except a dull aching of about two inches in breadth on the right side of the spine, extending from the lower part of the neck to the sacrum, corresponding with the situation and course of the longissimus dorsi muscle. The pain above the hip now began to subside, and in the space of three minutes from its commencement, had ceased altogether.

" 'The uneasiness along the course of the spine still remaining, a needle was introduced about an inch from one of the upper dorsal vertebræ, and another in a corresponding situation to one of the lower lumbar vertebræ. The pain in the right side was in a few minutes entirely dissipated, and the patient arose, declaring that, excepting a slight degree of uneasiness on the posterior part of the chest, near the angles of the inferior ribs, he was completely relieved from the disease. He, however, requested I would pass a needle in this last situation; on effecting which the pain soon left its last refuge, and the patient dressed himself, and left his house in the most perfect health. I have this day seen him, and he assures me that he has not experienced any return of the affection."

We shall conclude our analysis with one short extract, descriptive of the operation.

" The handle of the needle being held between the thumb and fore finger, and its point brought into contact with the skin, it is pressed gently, whilst a rotatory motion is given it by the finger and thumb, which gradually insinuates it into the part, and by continuing this rolling, the needle penetrates to any depth with facility and ease. The operator should now and then stop to ask if the patient be relieved; and the needle should always be allowed to remain five or six minutes before it is withdrawn. This mode of introducing the needle, neither produces pain (or at least very little) to the patient; nor is productive of hæmorrhage, which Dr. Haime says arises from the fibres being separated, rather than divided by the passing of the needle; the former of which (the absence of pain) is a point in its favor, which few surgical operations possess." 81.

We have now brought forward sufficient matter to excite the attention of our surgical brethren towards the publication of Mr. Churchill, and the operation of acupuncture—all that can be expected of us in this stage of the business.*

8. *Renal Calculi.*† Mr. Earle differs in opinion from Dr. Marcet and most recent writers on this subject respecting the formation of calculous matter, and suggests the

---

* The needles may be had at Mr. Blackwell's, Bedford Court; and Mr. Laundy's St. Thomas's Street, Borough.

† Mr. H. Earle. *Med. Chir. Trans.*

probability that, in some instances, the process may depend on a local morbid action of the kidney, independent of any predisposing constitutional cause. In support of this opinion, several important cases and dissections are adduced. We ourselves have long suspected that the disposition to form urinary calculi has been often superinduced by external injuries in the region of the kidneys; particularly in those who void gravel only after riding on horse-back, or from the motion of a carriage. Analogy would lead us to suppose that the calculi are generated under these circumstances by an inflammatory process, which we know has the effect of retarding or suspending the specific functions of secreting organs, and of favouring the production of apparently new formations.

The treatment suggested by Mr. Earle for this species of gravel is judicious, and appears to have been beneficial in one or two instances.

"In all instances where the affection can be at all traced to local injury, we should combine local with general treatment, and endeavour to arrest the morbid action by the abstraction of blood, warm bathing, and counter-irritants. Such a practice might perhaps be advantageously adopted in other cases not arising from injury, particularly where only one kidney appears to be affected; or, although the disease may have originated from constitutional derangement, it is highly probable that the local diseased action may be kept up after that has subsided, particularly should there be any calculus in the kidney too large to pass by the ureter." 227.

The counter-irritation Mr. Earl prefers, is that produced by a seton.

9. *Colchicum Seeds.** Dr. Williams, with laudable zeal and unwearied industry, continues his observations on the seeds of Colchicum autumnale—on the proper time for gathering and preserving them—on their superiority over the root of the plant—and on their efficacy in subduing chronic rheumatism, and other painful diseases. As the contents of Dr. Williams's valuable paper are now sufficiently diffused through the profession, we merely allude to them here, for the purpose of informing our readers that the objection we urged in our review of Mr. Haden's work, in our last number—namely, the *scarcity and dearness* of

* Dr. Williams. *Med. Repos. June,* 1821.

the Colchicum seeds, is now substantially removed. We have received a letter from Mr. FITCH, CHEMIST, of IPSWICH, with some specimens of the Colchicum seeds, gathered at different epochs, which we have distributed among our friends for forming the *vinum sem. colchici*,* and employing it in their practice. Mr. Fitch has gathered between one and two hundred pounds of the seeds, and, in a short time, expects to collect nearly a ton weight of them. Those gentlemen, therefore, who are anxious to employ the remedy, can procure it from Mr. Fitch at a very trifling expense—we believe not more than seven or eight shillings per pound.†

10. *Extraordinary Case of difficult Labour*.‡ We notice the following case, because we have often wondered that similar cases do not happen oftener.

On the 24th October, Dr. Battzell was sent for to Mrs. N. who had been in labour two days. The liquor amnii had passed off with the first slight pains, and, as the head of the child did not advance, the family physician was called in. The presentation was found to be natural; but the pains had now ceased altogether, and the patient had had a convulsive fit. On examination, Dr. B. discovered the os tincæ fully dilated, and recommended the application of the forceps. But repeated attempts with this instrument proved fruitless.

"Being satisfied that the delivery was impracticable with instruments, I introduced my hand, with a view of turning the child and delivering by the feet. But to my utter astonishment, I reached a part so tightly enclosed with the uterus, that with the utmost exertion of my hand, I could not pass my fingers between it and the body of the child. It then also appeared to me, that, when the head seemingly yielded to the force of the forceps, it was the uterus with its contents, that was drawn down towards the pelvis, and what surprised me, after all these attempts, the contraction now extended itself in an increased degree to the os tincæ. I then suggested the abstraction of blood by copious venesection to relax the system, and to embrace the moment of its immediate effect to extract the fœtus with the forceps. This also failed.

* See the formula in No. 4 of this series, p. 769.

† At our suggestion, Mr. Fitch has transmitted a quantity of the Colchicum seed to a house in London, [Mr. S. Fitch, at Messrs. Taylors and Martineau, Red Bull Wharf, Upper Thames Street,] where it may be procured in abundance, and depended on as properly preserved.

‡ In a letter from Professor Hall, of Baltimore.

On the following morning, bleeding was resorted to a second time, but with the like failure in repeated efforts. From manual examination and combined circumstances, I inferred the case to be what is usually termed 'the hour-glass contraction' met with sometimes in the retention of the placenta, but, never heretofore apprehended by me, as a cause of failure in delivery." *Medical Recorder, No.* 14, *p.* 215.

Convulsions now recurred repeatedly, and that evening the unfortunate woman expired. This lady was in the prime of life, of low stature, extremely fat and corpulent, and this was her first child.

On dissection, the uterus was found elongated, forming a tight case over the breech of the child. On opening into this part, some offensive effluvia issued forth. The uterus was closely contracted on the body of the child, presenting inequalities correspondent to its shape.

" Near the neck and shoulders of the child, the uterus had the appearance, as if a circular band, about half an inch wide, had been forcibly drawn around it. The stricture was accompanied with a prominence on each side, forming as it were, a groove in its course around that part of the uterus. It was this which I felt in endeavouring to pass my hand over the back and shoulders of the child. This fixed and forcible contraction, as with a cord, bound fast the fœtus and presented an insurmountable barrier, defying all the efforts that were used at delivery. The fœtus lay in its natural position with its breech uppermost, the knees bent and feet drawn close to it. It was a male child, large and perfect in its structure. P. 216.

Our author queries what would have been the effect of abstraction of large quantities of blood, administering at the same time the ergot of rye ?

11. *Arteritis.** Mr. B. Golding and the editor of this journal have lately attended an exquisitely marked case of *arteritis,* accompanied with *hypertrophia* of the heart. A considerable proportion of the symptoms enumerated in the article " *arteritis*" in our last number, were present. The patient had neglected his complaint till the disease, especially of the heart, had made great progress. Mr. Golding will probably publish the case. Dr. Johnson did not see the patient till two days before his death. The prominent symptoms were, very laborious respiration, not affected by

* See Art. 4 of last number, page 50.

position—violent and diffused action of the heart—intense throbbing of all tangible arteries—pulse excessively hard; about 105—great anxiety and restlessness—no sleep—thirst —very little cough—tongue clean—bowels deranged. The blood drawn at this late stage was remarkably buffed and cupped; but little or no alleviation was produced.

On dissection the lungs were sound; adherent on the right, but free on the left side, with about a pint of reddish serum, effused there. The heart was considerably enlarged, and entirely empty of blood. The parietes of the left ventricle were an inch and a half in thickness, but the cavity not at all dilated, constituting the hypertrophia simplex of Laennec. The right side of the heart did not seem affected. The internal tunic of the aorta and all its branches, carotids, brachials, iliacs, and femorals, was red as crimson, and all the blood in the body was fluid and thin. No other organ than the heart and arteries was affected. The man was about thirty years of age; had been ailing for eighteen months, but only two months what he called ill. He was about a week confined to bed. The pulmonary artery and the veins of the body were unaffected. The inflammation was confined to the aortic system.

12. *Diseases of the Brain.** To ascertain the actual seats of diseases is rendered extremely difficult, in consequence of their manifestations being often at a great distance from their original and fixed abodes. The play of the sympathies enables maladies to appear before us *en masque*, and thus mock our most sapient conclusions. In fact, they verify to the very letter, the fable of Proteus. The following case will sufficiently illustrate these observations :—

Theodosius, a young man, 24 years of age, of an excellent constitution apparently, and who had never been ill, complained, in Jan. 1815, of feeling slight giddinesses in the head, and momentary indistinctness of vision, accompanied by a sense of oppression on the chest, and a check on taking a deep inspiration. Mean time, the tongue became foul, the appetite impaired, and the digestion difficult. To these symptoms succeeded nausea and vomiting, at uncertain, but shortening intervals. The gastric irritability quickly increased, so that by the 1st February no aliment would lie on his stomach. At this epoch, an emetic was prescribed, on the dangerous principle —*vomitus vomitu curatur*. The patient brought up much bile, and

* Journal Gen. de Med. No. 289.

a large lumbricus; but no abatement of the symptoms followed; on the contrary, the gastric irritability was much increased. Opium was tried—a Burgundy pitch plaster, *with emetic tartar*, was applied to the epigastrium, and afterwards a blister to the same part, while the patient was supported by glysters of broth, and immersed in the warm bath twice a day. But all was in vain, for he expired on the 8th of February, never having complained of pain in the head, till the morning of the day on which he died.

*Dissection.* Four or five fungous vegetations on the superior part of the dura mater—adhesion of the dura mater to the pia mater opposite to these excrescences—ossification commencing in the falx major—six or seven ounces of water in the left, and four or five ounces in the right lateral ventricle—the sinuses and vessels of the brain gorged with blood. The liver was gorged with blood—and there was slight phlogosis of the mucous membrane of the stomach.

That this young man died of inflammatory action there can be no doubt—and that no proper means were taken to prevent the fatal catastrophe, except the low regimen, there can be as little doubt—at least, in this country. English physicians may sometimes carry depletion beyond the normal point; but they do not allow their patients to slide out of the world on mucilage of gum arabic and *eau sucré*, without making an effort to save them.

13. *Injuries of the Bones in Children.** We have seen several instances where the bones of children were *bent* by external violence, and some deformity produced from the nature of the accident not being properly understood. In such cases there is no crepitus nor actual fracture, yet they require the same management as fractures. This accident has been noticed by Boyer, Aitkin, Underwood, and some others, but the treatment has not been pointed out. It most frequently occurs in the bones of the fore-arm, in children of two, four, or six years of age. The symptoms are, pain and loss of power in a limb—a preternatural curve, which can be diminished or increased at pleasure, the bones, when straightened, however, having a disposition to return to their flexed position, unattended by any crepitus, or that circumscribed projection which a displaced fragment of bone produces. The proper treatment is to straighten the limb, and apply splints, in the same manner as in a common fracture.

* Dr. Barton.—*Amer. Med. Recorder, vol.* iv.

14. *Secale Cornutum.** In a late number of our esteemed cotemporary, the AMERICAN MEDICAL RECORDER, Dr. Atlee of Philadelphia renews the attention of his professional brethren to the *ergot*, or *spurred rye*, considered by Dr. Tully, who has written largely on the subject, to be a *parasitic fungus*, like the different sorts of blight, smut, &c. This medicine appears to exert a specific power on the uterus, causing and increasing the contractions of that organ, not only when there is a predisposition to action therein, but when a relaxed state of it requires that these contractions should be excited. Dr. Atlee's experience enables him to subscribe to the following indications for its use, viz.

"1. In the early stages of pregnancy, when abortion threatens, and has withstood the usual remedies of venesection, opiates, internal and external refrigerants and styptics, when the hæmorrhage is alarming, and the contraction of the uterus feeble.

"2. In cases of hæmorrhage near the end of gestation, when contractions have either not taken place, or are too weak to be effectual.

"3. In lingering labours, connected with the death of the child, or in such as are rendered so by a cessation of contraction, the os uteri being well dilated, and a proper relaxation of the other soft parts existing." 142.

A case occurred to our author where the labour had so far advanced that the membranes were ruptured, when the pains entirely ceased, and did not return during the whole night. The ergot was now administered in the dose of a scruple; and in half an hour afterwards she was delivered. He thinks this medicine essentially useful in labours rendered difficult by the too great comparative size of the head, or narrowness of the pelvis not amounting to deformity, and where the pains, though powerful, only serve to push the elongated scalp into the interior strait, where it may rest for hours, and ultimately require the forceps.

In uterine hæmorrhage, our author has exhibited this medicine with much success. In a peculiar condition of the uterus also, which sometimes takes place, *post partum*, namely, a torpor of the organ after difficult labour, preventing the contraction of its fibres, the womb remaining for hours, or even days, distended, hard, and attended with painful cramps, the ergot gave quick relief.

In a subsequent number of the same Journal Dr. Shallcross has inserted a paper on the efficacy of ergot in uterine hæmorrhages. He observes that—

* Dr. Atlee of Philadelphia. Dr. Shallcross.

"The effect of ergot in exciting unremitting contraction in the uterine fibres, renders its agency peculiarly suitable to cases of uterine hæmorrhage, which are generally produced by a partial separation of the placenta. The circulation of the blood through the vessels of the uterus, is impeded during the continuance of contraction, and the reciprocal pressure of the placenta on the child and parietes of the uterus, will favour coagulation in the orifices of the bleeding vessels." 219.

The first case which he details was that of a woman in the 42d year of her age, and in the ninth month of her fifth pregnancy. The flooding had commenced, without apparent cause, three hours before Dr. S. arrived, and was still considerable. Her strength was much exhausted, and the undilated state of the os uteri formed an objection to the introduction of the hand to effect delivery by the feet.

"The plan adopted was as follows: The woman was placed in the best possible position to preserve her from syncope, and the vagina stuffed with some soft tow and part of a flag handkerchief—one scruple of ergot was then administered in substance. In fifteen minutes the pains, which had before been slight and irregular, came on with considerable energy; the substances placed in the vagina were now carefully removed; the os uteri was found about the size of half a dollar, with its edges thin and the membranes spread tense over it. With a view to accelerate delivery, the membranes were pierced and the liquor amnii discharged; and in thirty minutes the child was born. There was no fresh hæmorrhage after the membranes were ruptured, nor in the delivery of the secundines. On the surface of the placenta were two clots of firmly coagulated blood, the size of an egg, flattened. The death of the child in this case cannot be attributed to any agency of the ergot." 220.

We have not space to extract more cases or observations. The foregoing will be sufficient to excite attention to the secale cornutum.

15. *Vacuity of Arteries, post mortem.** It was the opinion of Harvey, and his followers, until very recently, that the blood is circulated by the combined agency of the heart and arteries; and that the heart continues to propel blood in the last struggles of life, after it has ceased to receive it. Mr. Ker, of Aberdeen, has, in a late publication, denied the doctrine of the circulation altogether, and be-

* Dr. Carson.

come the strenuous advocate of the opinions of the ancient physiologists. Dr. Carson's theory of the motion of the blood has been some time before the public, and a full explanation of it may be seen in the first volume of the Medico-Chirurg. Journal and Review, page 515. In the second volume of the same Review, our readers will also find some illustration of this subject by Dr. James Johnson, in a correspondence with the elder Dr. Parry, at page 358.

In the present paper, Dr. Carson has published some experiments on animals to prove—

> "That the difference of the distribution of the blood after death from that in which, according to the Harveian theory, it must exist in the living system, arises chiefly from the elastic power of the lungs; and that the emptiness of the arteries and of the smaller vessels observed after death, admits of a satisfactory explanation from the supposed operation of this cause, combined with that of the elasticity of the arterial canals." *Med. Chir. Trans. Vol.* xi. *p.* 180.

In these experiments the collapse of the lungs was effected by making an opening through the muscular part of the diaphragm on each side, which was accompanied with the sound of air rushing through the orifices, and followed by speedy death. The vessels of the intestines, stomach, and mesentery, were very distinct and full of blood. The flesh was remarkably red, and, when cut into, bled. The heart and vessels about it contained only a moderate quantity of blood. A part of the descending aorta, above the iliac bifurcation, after its extremities had been secured, was cut out, and found to contain a small cylinder of blood generally coagulated.

These appearances are not exclusively the result of experiment: they have been met with after sudden death occasioned by an epileptic paroxysm. In the medical observations and inquiries will be found an instance of this kind related by Dr. W. Johnstone, in which the left ventricle and the aorta were found on dissection to contain nearly as much blood as the right ventricle and the cavæ. The sinuses of the brain were nearly empty, and the arteries were so turgid, that the drops of blood, which came out in great abundance, upon cutting into the substance of the brain, were also larger than usual. Dr. J. observes that Dr. Short had known a rupture of the left ventricle produced by the violence of an epilepsy. In Dr. Johnstone's case the lungs were of their natural colour and spongy; and the hands and feet were of an intensely livid colour, while every other external part of the body was pale. Hence he supposed that the congestion of the arterial system arose from the pressure of the convulsed muscles, or a spasm of the arteriolæ minimæ. In the works of Bonetus and Morgagni are also to be found instances of aortal congestion and of coagula, formerly called polypi, in the left ventricle; while the right heart, and the cavæ, were comparatively collapsed.

We admire Dr. Carson's theory of the circulation, and think that the society have shewn their judgment in publishing his paper, which will be an acceptable treat to the profession. His physiological researches have stamped a credit on his zeal and talents, which will insure the good opinion of his medical friends and the confidence of the public; and we are happy to find him placed in a situation, where his abilities will not fail to be duly appreciated.

16. *Obscure Uterine Disease.** A married woman 45 years of age, had never borne children, but evinced no sign of uterine obstruction till about two years previous to her final illness. In 1817 Dr. Mitchell was consulted, in consequence of the discharge of an obstinate fluor albus having become of a pink colour. This yielded at length to the tincture of lytta. The lady now enjoyed good health for a considerable time. In 1820 she discovered a hard tumour in the right iliac region, which she neglected to make known for several months.

"She was frequently seized suddenly with severe paroxysms of pain in the region of the uterus, attended with vomiting, breathlessness, numbness of the right thigh, and much difficulty in voiding the contents of the bladder and intestines." *Med. Recorder, p.* 137.

False delicacy prevented an examination, per vaginam, till a short time before her death. Before the tumour had advanced much in magnitude, evident relief was obtained by rest in the recumbent posture. The appetite was generally defective. In the intervals she had tolerably good health and spirits. Her last paroxysm lasted two weeks, and had been brought on by exposure to wet.

"Her nights as well as her days, were restless, her whole frame was racked with pain, her stomach almost incessantly contracted with fruitless efforts at evacuation, and the soft parts in the lumbar region burning with a heat that nothing could alleviate. Towards the closing scene, she was occasionally delirious, and on the day

* Dr. Mitchell, of Philadelphia.—*Med. Record. No.* 13.

preceding her death, became so exhausted as to be unable to converse, or help herself in bed. This exhaustion, however, was attended with considerable mitigation of suffering, and gave to her last hours comparative ease. About an hour before she died, she was conscious of something bursting within her, after which the tumefaction extended to the epigastric region, increasing the vomitive efforts, and breathlessness, and finally terminating in death." 137.

Such is the history of the disease; and as no dissection was permitted, the exact nature of it must be a matter of conjecture. The only medicines that gave any alleviation was a diuretic, in which squill and iron formed the principal ingredients. Externally hot spirituous fomentations were also serviceable. Two days before death an examination, *per vaginam*, was permitted; but this only rendered the disease more obscure, in the eyes of the medical attendants.

"Low down in the vagina, was felt a large round tumour of great firmness, pressed down into the pelvis almost as firmly as the head of a child in the advanced stage of labour. This was of course the uterus, but the os tincæ was altogether wanting. It appeared to me a firm tumour or thick sac filled with some sort of matter, but having not the smallest aperture. On passing the finger up towards the right os ilium, it was resisted by what appeared to be adhesion of the tumour; on the left side, the finger could be passed rather higher up." 139.

The result of this examination was a belief that the disease was either an enlargement of the uterus, or that it was "converted into a thick tight sac containing blood, pus, or some other matter." This case will afford speculative materials for the accoucheur.

17. *Ulcer in the Stomach.** The following case, bearing no trifling analogy to that of the once renowned, but how nearly forgotten, Napoleon Bonaparte, we shall give in the words of the author.

"On the 1st of June, 1819, a gentleman about sixty years of age, who had led a remarkably regular and temperate life, was attacked with a peculiar uneasiness in the abdomen, which was thought to arise from irregularity of the bowels. A variety of evacuant medicines were used, but gave him no relief. Fomentations and anodyne embrocations procured only temporary benefit. He continued in

* Dr. Cheeseman, New York.

this state for about a month, with little or no variation, when he was suddenly seized with a violent pain in the superior part of the rectus abdominis muscle, extending down to the umbilicus, and inclining to the left side. In consequence of the violently spasmodic affection of this muscle, a variety of anti-spasmodic medicines were directed, but with very inconsiderable advantage. Bloodletting, warm bathing, &c. were productive of no better effect. It may be proper to state, that this gentleman had, for several years, suffered occasionally from a rheumatic affection of the right leg, thigh, and hip, which had produced considerable lameness, and greatly impaired his general health. The disease under which he now laboured, was thought to be of the same nature; and as all the ordinary remedies had failed in affording alleviation to his distress, he was advised to try the effect of sea-bathing. This remedy was faithfully had recourse to, but without any advantage to the local affection, or any improvement to his general health. He was gradually exhausted by this continual irritation, and died on the 30th of August, 1820." *Med. Rec.* 152.

On dissection, an ulcer was found in the stomach, "about three inches in diameter, the edges of which were high and much jagged." Intus-susception was found in several portions of the intestinal canal.

18. *Panckouckerie.* We have always ceded the palm of victory, in respect to fertility of invention, to our Gallic neighbours. A Parisian bibliopolist, M. Panckoucke, has lately outstripped all competitors in the art of picking the pockets of the public throughout Europe, and that with their eyes open, and in the broad face of day! Some ten or fifteen years ago this gentleman issued the prospectus of a Dictionary of Medical Sciences, edited by the most eminent men of the age, and which was to be composed of choice and select monographs, containing all that was good, and rejecting all that was false or doubtful in medical science and literature. What a delightful prospect! Every one opened his purse, and subscribed his twelve francs in advance. The work was to be comprised in twelve volumes —but as that was found insufficient, 18 volumes were to be the extent. Arrived at this limit, they had only got to the letter G. Well! 24 volumes would certainly finish. But the 24th volume only got two letters beyond the 18th!! In short, we have now 53 volumes, and we have only got to the letter S! But this is the least of the evil. This immense tree must push out branches corresponding to the trunk. Accordingly we have an extra series of Flora's, amounting already to 90 numbers; and to make the Flora complete, we must have an "*iconography*," of 17 numbers to give employment to the pretty fingers of Mrs. and the Misses Panckoucke! This subject being likely to fail, another branch or series was invented—*biography*, which will occupy 12 or 14 volumes extra! But the *chef d'ouvre* of invention still remained. Other dictionaries contain but 24 letters. M. Panckoucke seized, with delight, on the *et cetera*, which is to have *no end!* The *Journal Complementaire des Sciences Medicales* is to form a tail to this literary comet, which is to blaze along the horizon, *per omnia secula seculorum.*

Still M. Panckoucke is not content. He has swelled out the Dictionary with a chaotic mass of useless and heterogeneous materials, among which a valuable article here and there is studded. He now is going to publish an *abridgment* of the whole work—not a selection of the good from the bad, but an indiscriminate *rasée* of each article; so that in the abridgment there will be nothing good to compensate for the trash.

Our friend, Dr. Ducamp, of Paris, has exposed, with great humour and truth, this literary pick-pocket, in the February number of the *Journal General de Medecine;* and we take this opportunity to warn our brethren on this side of the channel against such unprincipled speculators.

Our readers are all aware that we take every opportunity of diffusing any valuable information which we find in the above work (and there are numerous sterling monographs) among the profession in this country, for the size and language of the original completely check its circulation in any other shape. This practice we shall steadily continue, and we hope to enrich our pages, from time to time, with the productions of some of the ablest of our brethren on the Continent.

19. *Ophthalmic Hospital, Regent's Park.** This expensive establishment is extinct, and the invidious appointment of a civilian over the heads of the army medical officers cancelled. The mighty claims to success founded on certain "peculiar operations," have dwindled down to the humble merit of having "been greatly instrumental in *promulgating* knowledge," which the committee traces up to Celsus, and down again to the reign of Queen Anne. As a

---

* Report of Select Committee.

reward, however, for Sir William Adams' four years' service in the Ophthalmic Hospital, the committee have recommended that the sum of four thousand pounds be granted him. We have understood that this sum is not above a tenth of what Sir William expected. But let him thank God and Lord Palmerston for what he has got!

We are informed that Sir William Adams stated to the committee that, as the whole of the medical periodical press of this country was conducted by, or under the influence of physicians and surgeons belonging to His Majesty's service, so his works were all misrepresented, and their extraordinary merits kept back from the public. All we shall remark on this is, that it is a desperate bad cause which requires mis-statements to support it. That pre-eminent medical talents, in all ages and countries, have been assailed by envy and jealousy, the history of illustrious individuals too fully proves; but we believe there is no instance on record where a general combination has been raised against any professional character, without the most solid grounds for such proceedings. In the present frame and constitution of society, it is morally impossible that such a combination should take place, and therefore Sir William Adams, in our humble opinion, acts a very impolitic part in making use of such a defence.

We can disprove Sir William Adams' statement by an appeal to facts which are within the view of all our readers. We gave a full and favourable analytical review of Sir William's work on Cataract, in the second volume of this Journal, long after the controversy between the army and himself had commenced; and still more recently we reviewed his work on artificial pupil, at the same time with that written by Mr. Guthrie, without making the slightest allusion to the controversy, or suffering ourselves to be influenced, in the most remote degree, by any feelings hostile to this man, who casts such a sweeping reflection on the whole periodical press. We leave Sir William Adams to reconcile these *facts* with his *assertions*, if he has any regard to *professional* opinion, which we firmly believe he *has not*—excepting as far as his own pecuniary interest is concerned. Sooner or later, Sir William Adams will find that the conduct which secures the esteem of his professional brethren is that which will most effectually and permanently contribute to his prosperity, as well as happiness. At present, he will, of course, laugh at us as not being "men of the world;" but let him bear in mind the words of a celebrated ancient—"mark the end."

20. *Masked and Periodical Intermittents.** Many of the enthusiastic followers of Broussais would appear to reject the agency of the *nerves*, or at least the state of *spasm* as an item in the proximate causes of disease. According to their doctrine, the *scalpel* alone is necessary in unravelling the intricacies of pathology—while leeches and low diet form the sum total of therapeutics. This is striking out a nearer cut to the summit of the hill of science than Brown ever dreamt of. In all ages and countries, physicians employed the lancet and other antiphlogistic measures, when they were indicated by the phenomena present, and before the light of morbid anatomy had risen on the horizon of medicine. But in all ages and countries, on the other hand, observation demonstrated that there was another class of diseases which could not be called, or treated as, *inflammation*, and which were obviously referrible to a state of the nervous system which, for want of a better, has been given the term of *irritation*. And if these affections of the nervous system were recognized in the earlier periods of civilization, they are still more numerous now, when the habits, moral and physical, of mankind, tend particularly to their multiplication.

But the Broussaians point to the numerous cases of recovery from (supposed) gastritis, or gastro-enteritis, by the application of leeches to the epigastrium or abdomen. Yet men who do not ride a hobby-horse will answer, that many of these pretended phlogoses were only states of irritation or spasm, which would have got well by quiet, low diet, and gentle means, had leeches never been applied:—that the greater number of these patients can afford to lose blood by leeching, without any inconvenience, be their complaint what it may; and moreover that local blood-letting is very often serviceable in reducing states of irritation, where not a particle of inflammation exists. At the same time it must be confessed that, in not a few cases, the needless abstraction of blood, where the disease was of the nervous or irritable character, although it may have produced a temporary mitigation, was followed by a general or local debility that either prolonged the present, or predisposed to other maladies.

Such are the reflexions of M. Comte, and we must say

---

* Des bons effets des antispasmodiques et principalement de l'opium, comparativement a ceux de quinquina, dans les fièvres larvées et les intermittentes periodiques. Par M. Compte.—*Journal Gen. de Med. No.* 291, *Fev.* 1821.

that they are not without foundation in reason and fact. We shall now proceed to some of the cases which our author has laid before the public.

*Case* 1. A young man, after a hearty supper, was seized next morning with vomiting of bile and aliments swallowed. Every evening afterwards he was attacked with feverish symptoms, and such a violent pain in his neck that one night he attempted to wound himself with a cutting instrument, his tortures were so insupportable: yet there was no appearance of swelling or redness inside or outside of the throat. After the paroxysm, the patient was left weak, the pulse small, the countenance pale, and the features altered. The Peruvian bark and valerian soon put an end both to the fever and local pain. These medicines, however, were assisted by nitre, camphor, and opium, the skin having once or twice become constricted, and the nervous system irritable, till the latter remedies were used.

*Case* 2. A youth, 13 years of age, having danced rather too long at a ball, was seized with shiverings, and a tremulous motion of the lower extremities, which lasted about an hour, and then left him in perfect health. Next day, at the same hour, the same kind of paroxysm returned; but the agitation of the limbs was very violent. A slight head-ache and trifling chills preceded the convulsive movements of the lower extremities. After an hour's duration the fit vanished as before, and left him without ailment. Bark, valerian, and camphor, were now given in the intervals of the paroxysms, but only with the effect of rendering them later in their accession. On the 21st day the bark was abandoned, and some trifling means were used, but the disease became aggravated, and accompanied with convulsive movements of the whole body. The bark, camphor, and valerian, again exhibited; and the succeeding paroxysm shorter and weaker. The next only lasted ten minutes, and the third four. There was no return for ten days, at the expiration of which the paroxysms was renewed, and again controlled by the same means. But the boy peremptorily refused to swallow any more bark, and the paroxysms, though gradually lessening in force and duration, assailed him every day for three months, when Nature triumphed over the disease.

Among the prejudices which our continental brethren cherish, and indeed pride themselves upon, is that of not administering arsenic—because, forsooth, it is a *poison*. If you ask them what bad effects they have *seen* result from its administration? they answer, that it *must* have bad effects from analogy, and therefore there is no occasion to try it. These are precisely the arguments they urge against purgatives—and particularly calomel purgatives, in peritoneal inflammation. They *must* irritate and inflame the bowels, and therefore they will not try such dangerous remedies.

*Case* 3. We shall only quote, or rather abridge, another case from M. Comte.

Master V———, aged 19, after bathing in a river several times, in the summer of 1820, was taken with malaise, sleeplessness, agitation, and convulsive movements in all his limbs, which continued for a fortnight, and yet without loss of appetite. A pain took place in the left side, which was removed by leeches; but subsequently returned, though in a less degree, in both sides. On the 26th of August, after a good forenoon, he was seized in the evening with chills, violent head-ache, delirium, extreme restlessness, and convulsive movements in the muscles of the face. In this state he jumped out of bed, upsetting and breaking every thing that came in his way, four men being hardly sufficient to secure him again in his bed. 27th, Quite well, appetite good; but in the evening a similar paroxysm as on the preceding day, but accompanied this time with a difficulty of respiration, threatening suffocation. These symptoms all subsided without medicine, for some etherial antispasmodics only aggravated them. Bark and valerian were now prescribed in the intermissions. But the paroxysms returned with as much violence as ever, for several days. Laudanum, castor, and camphor, were now combined with the cinchona and valerian, and the accesssions were soon checked and stopped altogether.

At the conclusion of the cases M. Comte makes some sensible observations, and some severe remarks on the Broussaian school, who see only phlegmasiæ in all kinds of fever, continued or intermittent. He protests loudly against blotting out *idiopathic* fever from the nosologic chart, in favour of symptomatic. In this country, as far as we can form an idea of the general sense of the profession, *idiopathic* fever is acknowledged to have an existence—and that very frequently.

20. *General Phlebitis, and partial Arteritis** Modern pathology has lately directed its researches, with zeal as well as profit, to derangements of the vascular and nervous systems. Every day we become more and more acquainted with the causes of symptoms and the seats of disease. This is surely the right road to successful therapeutics. Facts, however, and accurate observations, under the existing regime of pathology, are wanted, and must be carefully recorded for centuries to come, before our science shall approach to any thing like certainty.

In the greater number of cases that are on record of the inflammation of veins, the causes have been *external;* but we believe the following presents the most extensive phlebitis, from a general or constitutional cause, that is to be found in the writings of modern physicians.

The patient was a batchelor, 38 years of age, who was seized, after a moderate fever, with distinct variola, on the 15th March, which ran its course regularly and mildly. During the eruption, the patient found himself so well, that he did not observe a sufficiently strict regimen in respect to food.

On the 22d March, the tenth day of the disease, the eruption being in full suppuration, the patient experienced, without any apparent cause, a general indisposition. He felt weak and fatigued—his limbs heavy and aching—his tongue yellow and coated—the breathing short—the pulse quick and hard. Hitherto he had taken nothing but ptisans. He was ordered a vomit. He only brought up some potatoes on which he had supped. From the 22d to the 25th, he continued in much the same state, the variola running its phases, but the pustules becoming rather flatter than is usual, and the areolæ of a dull red colour. The fever, though mild, persisted, and was accompanied with watching, and pains in various parts of the body. The appetite was entirely gone, the tongue foul, the breath fetid. On the evening of this day, he complained of an indefinable sense of sinking and lassitude. The eye exhibited a peculiar expression of dejection and suffering. The pains in the arms, thighs, and joints, now became so excruciating that the patient could not rest an instant in one position. He was excessively apprehensive for the night—thirst inextinguishable—tongue brown and dry—breath very offensive—pulse very wiry. At two o'clock in the morning the agitation declined—he ceased to complain, and, at six o'clock, expired.

*Necroscopia.* The variolous eruption entirely dried up—œdema of the right upper and left inferior extremity—a small suppurated phlegmon on the left elbow. *Head.* The arachnoid vessels much injected—a gelatinous layer spread over the right hemisphere of the

* Dr. Fallot. *Journ. Comp. July,* 1821.

brain—brain itself sound—no fluid in the ventricles. *Thorax.* The lungs gorged with black, fluid, and watry blood—mucous membrane of the bronchia and trachea injected. *Abdomen.* The peritoneum healthy—liver enlarged, and gorged with black blood—gastro-intestinal mucous membrane healthy. *Vascular System.* Heart of natural size and appearance externally—internal surface of cavities intensely inflamed throughout, but more so in the right than left chambers—the arches of the aorta and pulmonary artery finely injected with blood—the pulmonary artery and veins of a deep purple red colour, as far as they could be traced—the internal tunic of the aorta, as far as the arch, was of the same colour; from thence the tint got lighter and lighter, until it became extinct. The inner membrane of the coronary arteries was inflamed throughout their extent. The interior tunic of the venous system was not only inflamed, but also thickened wherever it was traced. The inflammation too, increased as the vessels receded from the heart—the branches being most, and the trunks of the veins least, inflamed. No washing, sponging, or even scraping with the scalpel could remove this inflammatory appearance. The lymphatic vessels were not affected.

M. Therion, a young eleve of the faculty of Liege, did not leave the body until he had opened almost every ramification of the vessels, of which he made some beautiful drawings. We need not remark on the inertness of the practice in this case, because it is not likely that any treatment would have succeeded.

